# The sensitivity and response of the threatened endemic shrub *Arbutus pavarii* to current and future climate change

**DOI:** 10.1186/s12862-025-02370-2

**Published:** 2025-04-24

**Authors:** Emad A. Farahat, Amel F. Tashani, Ahmed R. Mahmoud

**Affiliations:** 1 Botany and Microbiology Department, Faculty of Science, Helwan University, P.O. Box: 11795, Helwan, Egypt; 2 Department of Forest and Range Science, College of Natural Resources and Environmental Sciences, University of Derna, P.O. QM27+3CP, Derna, Libya

**Keywords:** Libyan strawberry, Endemic species, Climate change scenarios, Al-Jabal Al-Akhder, Modeling

## Abstract

Climate change is expected to significantly alter and modify the ecological conditions of plant distribution and growth, particularly in the Mediterranean Basin, which is considered one of the hot spots for global warming. Measuring and modeling the response (sensitivity) of wild plants to current and future climate is critical to predicting future biodiversity and ecological values. *Arbutus pavarii* Pamp. (family Ericaceae) is a narrow endemic Libyan medicinal plant and one of the Red List species according to the IUCN that faces the threats of extinction due to habitat deterioration, overuse, and low reproductive rates. In this study, the species distribution model (SDM) approach was used to model and forecast range shifts in *Arbutus pavarii* under current and future climate change scenarios at various Shared Socio-economic Pathways SSP1-2.6 (lowest emission scenario) and SSP5-8.5 (highest emission scenario) for the years 2050s and 2070s. The modeling results indicate that the current highly suitable areas of the plant will decrease in the future compared to the low and moderate ones. The distribution range of *A. pavarii* will increase under lower emission scenarios (SSP1-2.6, 2050s) by 1.12% but under higher emission scenarios (SSP5-8.5, 2070s), the suitability of the habitat will decrease by 1.39%. Given the low reproductive fitness and the anticipated rise in air temperature, *A. pavarii* is likely to encounter greater challenges in its natural existence and dispersal. Lands with high elevation and precipitation are suitable for its future distribution. We recommend further ecophysiological and tree-ring studies on this species to investigate its growth-climate relationship and performance under drought conditions. The in-situ conservation of *A. pavarii* as well as its cultivation in the projected high and moderate habitats are recommended. Local community engagement may be beneficial in any conservation program for this species.

**Supplementary Information:**

The online version contains supplementary material available at 10.1186/s12862-025-02370-2.

## Introduction

The Mediterranean basin is characterized by cultural, landscape, and biodiversity richness. It is also a region of constant change due to exponential human activities, industry, and population growth [54]. The Mediterranean basin is known to be one of the most vulnerable areas of the world to climate change [37]. Since the 1980s, the aridification trends in this region have become worse and there are more negative impacts on the forests’ productivity [52]. According to a projected 40 percent less precipitation scenario during the winter rainy season, it is expected that the climate of the Mediterranean basin will be drier than any region on Earth [54]. This well-observed decline in rainfall will lead to negative impacts on the growth responses of tree species to climate warming. According to Farahat and Gärtner [24], the Mediterranean basin witnesses an exceptional increase in air temperature, a decrease in annual precipitation, and drought frequency. Dai et al. [12] and Russo et al. [51] reported that strong drought events were prevailing in the Mediterranean basin during the second half of the last century. Severe weather events (e.g., heat waves and drought) have significant negative effects on forest productivity and net primary production in North Africa [10, 55].

**Fig. 1:**
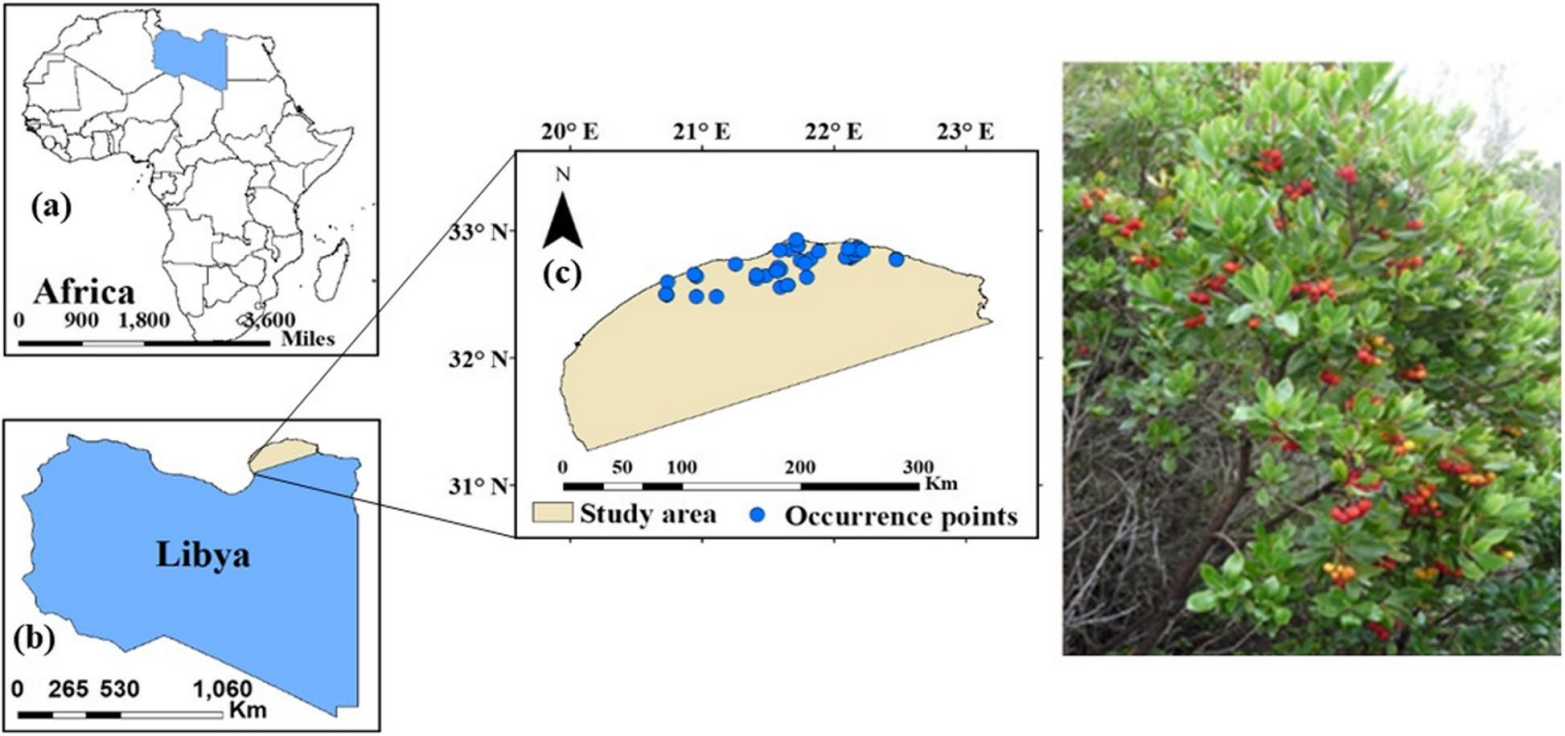
**a** Africa map showing the location of Libya, (**b**) Libya map declare the study area location, and (**c**) Study area (Al-Jabal Al-Akhdar region) with occurrence points (green circles) of *Arbutus pavarii*. An image of the plant during the flowering stage is shown

Cyrenaica is considered the largest phytogeographical region In Libya. This includes the Al-Jabal Al-Akhdar (the Green Mountain) region (AAR) in the northeast of Libya, which is the richest vegetation and highest species diversity landscape in Libya [34] Fig. 1. The AAR has considerable potential for agricultural activities since natural resources are promising. Among the plant diversity in the AAR ecosystem, the species in the AAR that are used economically and medicinally cover about 500,000 ha, around one-third of which have been converted into growing crops. The actual area of the AAR productive forests is about 320,000 ha [6]. *Arbutus pavarii* Pamp. (family Ericaceae, commonly known as Shmar, Shemri, or Libyan Strawberry) is a large shrub or small tree, and a narrow endemic Libyan medicinal plant [39]. It is located in dry to subhumid bioclimate, on the thermo-Mediterranean floor, with mild winters. It is classified as a near-threatened species according to the IUCN Red List [31, 38, 43]. The species is exposed to overgrazing and excessive human disturbance and is used for medicinal and wood fuel purposes [32]. The flowers *of A. pavarii* are an important economic source of pollen grains and nectar for bees [16]. Locally, the species is used for the treatment of gastritis and renal infections [17, 35]. El shatshat and Elshibani [15] found a high content of antioxidants, vitamins A, E, and C, and phenolic compounds in the ripe and unripe fruits of *A. pavarii*. Elshibani et al. [20] isolated fourteen compounds from the aboveground parts of *A. pavarii* using gradient solvent fractionation. Based on its chemical and biological profiles, they recommended *A. pavarii* to be used in some food production industries in the Mediterranean basin. The leaves of the species are a reliable source of antioxidants for industrial and food purposes [33]. The results of Ali et al. [5] revealed that *A. pavarii* species has antioxidant effects that can prevent human platelet aggregation.

Kabiel et al. [39] reported a significant positive relationship between the percentage cover and the reproductive output of *A. pavarii* in the AAR on the one hand and increasing in elevation otherwise. They reported the absence of new seed recruitment or production of new fruits in some populations of the species in their habitat. Mosallam et al. [43] reported that in the condition of extreme summer drought, *A. pavarii* does not produce flowers and seeds. This represents serious demographic threats to the persistence of the species. Nevertheless, elevation was not the main key factor that controls the density and size structure of *A. pavarii* populations. The occurrence of *A. pavarii* was restricted to a few valleys in the AAR and along the southern part of the mountain [34]. Abdalrhim [1] reported that *A. pavarii* dominates the clay depressions of the Al Marj-Al Baida motorway, while Hashim et al. [32] reported a high correlation between the soil clay content and the species distribution in the AAR. Due to habitat deterioration in the AAR, Harvey-Brown [31] and Santis et al. [14] observed an exceptional decline in the occurrence record map of *A. pavarii*. As a result of the species' declining habitat quality, limited biological range, and low recruitment rate, *A. pavarii* has become a threatened endemic species in Libya.

Species distribution models (SDMs) are used in ecology and natural history based on gradient modelling and ecological niche theory. The premise of these models is that environmental factors determine the distribution of species and consequently communities [29]. The main applications of SDMs focused on improving models that predict the response of species to environmental gradients [9], where modelling algorithms are employed on species occurrence data and environmental predictors to predict the species distribution over time and geographic space. The MaxEnt modelling approach is used to predict and estimate the geographical distribution of a species in a certain area. This is achieved by using current data on species occurrence together with the environmental factors that influence their spatial distribution [48]. The MaxEnt is recognized as one of the most accurate and high-performance modelling methods for predicting species distribution [19], especially when presence-only occurrence data are available and in cases of species with limited records of occurrence data [27]. The MaxEnt approach was chosen for this study due to its reliability, accuracy, and ability to predict species' future habitat suitability under climate change scenarios. It uses species presence-only data, both quantitative and qualitative environmental variables, produces a spatially explicit map, allows repeated runs of the model for robustness, and can be used for planning conservation measures [19].

Many studies used MaxEnt to predict the potential impact of climate change on the distribution of endemic and threatened species and habitat suitability under different scenarios. For instance, For the years 2050 and 2070, Hosseini et al. [36] applied it to *Thymus* species in Iran under two representative concentration scenarios (RCP 4.5 and RCP 8.5). Liao et al. [41] used it to predict the changes in the range of the endangered species *Encalypta buxbaumioidea* in China under three concentration scenarios for the years 2050 and 2070. The same was true for many other rare and endangered species in the world [21, 25, 28].

To our knowledge, there is not any published study dealing with the current and future responses of *A. pavarii* to the projected climate change. Therefore, to help in the conservation of *A. pavarii*, the present study aimed to: (i) identify the most contributing environmental factors to the species distribution, (ii) determine the suitable habitats for the *A. pavarii* distribution under the climatic conditions at current; (iii) assess the potential future effect of climate change based on HadGEM3-GC31-LL general circulation model for two Shared Socio-economic Pathways (SSPs); the 1–2.6 and 5–8.5 SSPs that assume different levels of emission scenarios between 2050 and 2070s horizons. We hypothesize that the results of the present work will be useful in determining the best locations for *A. pavarii* to survive and will be used for future conservation, particularly in locations where the population is losing ground.

## Materials and methods

### Study species and habitat

The present study area is about 42,156 km^2^ which covers the current distribution area of *A. pavarii* in Libya located between 32° 56′ 27.49" N and 31° 16′ 21.49" N, and between 19° 55′ 15.50" E and 23° 11′ 44.49" E (Fig. 1). Its distribution is restricted only to the AAR in Libya. The AAR is a mountainous highland along northern eastern Libya. It is a crescent-shaped ridge with an elevation of > 850 m a.s.l. at its central part (min. = 223 m a.s.l., max. = 876 m a.s.l.). The northern part consists of a step-like plateau bordered by escarpments. The southern part dips gently towards a depression extending from Ajdabiya to AI Jaghbub, which is marked by several large sabkhas. A coastal plain is well-developed between the foot of the first escarpment and the sea. The AAR is considered the wettest region in Libya, largely owing to its proximity to the Mediterranean and its altitudinal character [4]. The rainfall is concentrated during the cool winter and drought occurs in the summer. The annual average temperature is 16°C and the annual average rainfall is 550 mm. January is the coldest month, while August is the warmest. Precipitation lasts from October to April, with a maximum rate in December and January. The annual precipitation average is 400–600 mm.

### Species occurrence data

Forty-three records of *A. pavarii* occurrences were gathered from field studies in Libya and the online GBIF database (Global Biodiversity Information Facility, www.gbif.org, GBIF Occurrence Download 10.15468/dl.rdt6yj.

### Environmental variables and selection

We downloaded 71 climate variables and one altitude variable for the current period, and we used ArcGIS version 10.8 to calculate the aspect and slope variables (Supplementary Table S1). The United States Geological Survey Dataset was the source of the elevation data (https://www.usgs.gov). The current climatic variables were downloaded from the WorldClim database (http://www.worldclim.org, [26]). The bioclimatic data were downloaded from the WorldClim dataset version 2.1 at 30 arc-seconds (1 km) spatial resolution. The dataset includes 19 bioclimatic variables, along with 36 monthly average values for maximum temperature (tmax), minimum temperature (tmin), and precipitation. The 'current climate' data from the WorldClim database represents the climate during the period from 1970 to 2000, and this information is widely used in modeling species distribution (e.g. [13, 42]).

The downscaled future global climate model (GCMs) of the Coupled Model Inter-comparison Project (CMIP6) was represented by the HadGEM3-GC31-LL general circulation model (GCMs) data for future climate scenarios that were downloaded from WorldClim 2.1 (www.worldclim.com). This model was chosen because it accurately shows the regional patterns of precipitation and temperature distribution and accurately reflects the current climate as observed. The IPCC's sixth climate assessment report (AR6) included temperature and precipitation data for four Shared Socio-economic Pathways (SSPs) that assume varying emission scenarios: the 1–2.6, 2–4.5, 3–7.0, and 5–8.5 SSPs, as well as 23 GCMs. Data for two SSPs (SSP1-2.6 and SSP5-8.5) and two time periods -the 2050s (average for 2041–2060) and the 2070s (average for 2061–2080)- were obtained from the WorldClim database.

To establish a model that avoids multicollinearity issues while maintaining better performance with fewer variables, we calculated variance inflation factors (VIFs). Variables with a VIF value greater than 5 were excluded due to their negligible contributions [30]. The VIF calculations were conducted using the “usdm” package [45]. Specifically, the vifstep and vifcor functions within the usdm package [44] were executed using R software version 4.2.1 [50]. The test resulted in 9 variables with VIF values less than 5 for each and used in the modeling process.

All data layers representing environmental variables were maintained at a resolution of 30 arc-seconds (approximately 1 km) and clipped to the spatial extent of the study area using ArcGIS software version 10.8.

### Model construction and evaluation

We performed the environmental niche modeling and projections using MaxEnt software version 3.4.1 [48]. The model outputs were verified by randomly allocating 75% of the occurrence data as training data and 25% as model validation data. The logistic format was chosen for the model's output data because it incorporates environmental data to evaluate the probability of occurrence as expected. The convergence threshold was 0.00001, and the algorithm ran for 5000 iterations and 1000 as the maximum number of background points. ArcGIS software was used to convert the outputs into a raster format for additional investigation. Threshold-independent receiver-operating characteristic (ROC) analyses were used to assess the model's prediction ability [48]. MaxEnt computed the area under the curve (AUC) to measure the predictive capability of the produced model. The scale recommended by Swets [53] was used to estimate the model’s predictive ability. Low accuracy is indicated by AUC < 0.5, pure chance by AUC 0.5, failure by AUC 0.5–0.6, poor accuracy by AUC 0.6–0.7, fair accuracy by AUC 0.7–0.8, good accuracy by AUC 0.8–0.9, and excellent accuracy by AUC > 0.9 (Bui et al., 2016).

Each variable's contribution to the *A. pavarii* habitat model was assessed using the fifteen-repetition Jackknife test. The results of the test show the amount of gain that can be obtained from each variable alone or all variables together. When a variable has a greater gain value, it contributes more to the species' distribution (Wang et al. 2018). The Jackknife test revealed that prec10(67.64%) is the most contributed variable for the *A. pavarii* distribution then bio2 (9.48%) followed by prec1 (7.82%), tmax5 (4.6%), Aspect (4.48%) and bio7 (4.12%). The final map of potential predictions for the species distribution ranges from 0 to 1 value. Four classes of habitat suitability were identified based on the probability of occurrence: high potential (> 0.651), moderately suitable (0.351 – 0.65), low suitable (0.141 – 0.35), and unsuitable (< 0.14) [11, 49, 56].

### Potential distribution of *A. pavarii* under future climate scenarios

To anticipate the degree of suitability of habitat in the future, in the IPCC report (AR6), the values of temperature and precipitation were processed for 23 of the GCMs, and for four Shared Socio-economic Pathways (SSPs); 1–2.6, 2–4.5, 3–7.0, and 5–8.5 SSPs that assume different levels of emission scenarios. In this study, the prediction of the future distribution of the studied species was conducted under the GCMs of HadGEM3-GC31-LL for the future periods of the 2050s (averaged over 20 years from 2041 to 2060) and 2070s (averaged over 20 years from 2061–2080) and two Shared Socio-economic Pathways (the SSP1-2.6 and SSP5-8.5). This was performed to depict the variations in the future projections by the GCMs and to have insights into the future possibilities of *A. pavarii* under different emission scenarios both in the short-term and the long-term.

The prediction results of the established models represent the occurrence probability for *A. pavarii*, with a range between 0 and 1, where 0 indicates the unsuitable area for the species and 1 indicates the optimal area for the occurrence of the modeled species. To obtain maps of presence and absence areas for the species studied, the continuous probability maps produced as outputs of the established models were converted into binary maps representing the potential presence and absence areas for each species. Additionally, to classify the study area in terms of its suitability for each of the studied species, the probability maps produced as outputs of the established models were classified based on the range of the probability of occurrence into four levels of habitat suitability, high (0.651–1), moderate (0.351–0.65), low (0.141–0.35), and unsuitable (0 – 0.14) based on modeling output threshold. The reclassification feature in the Spatial Analyst Tools within the framework of ArcGIS 10.8 was used to perform the classification.

**Fig. 2:**
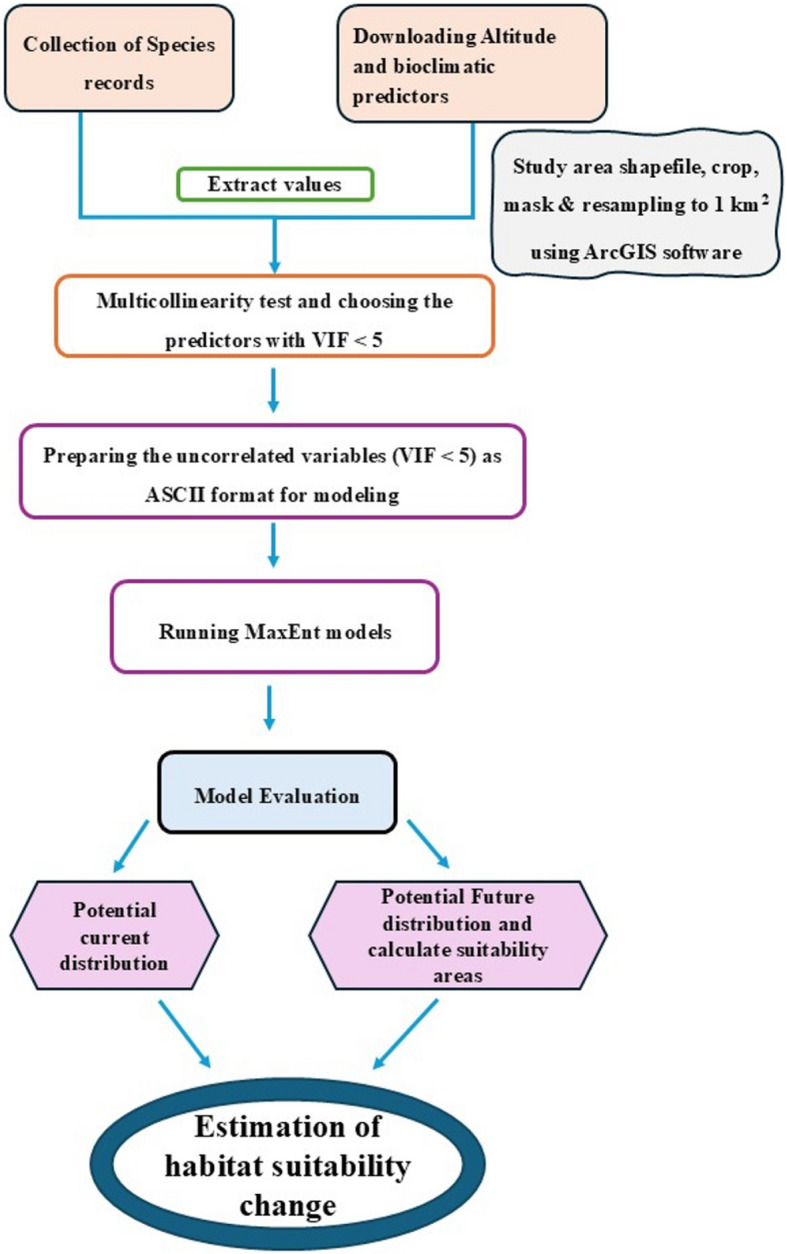
Flow chart shows the main steps in the modelling approach used in the present study

To measure the change in the distribution of the suitable habitat in terms of loss/gain or persistence under future scenarios, the functions within the framework of ArcGIS 10.8 were applied to binary maps resulting from the transformed current and future habitat suitability probability maps. The estimation of the changes in the distribution of suitable habitat between the current and the future scenarios in each period was calculated by SDM toolbox v2.5 within the framework of ArcGIS 10.8, and the potential change maps for each period under different scenarios were obtained. Through the estimation of the changes in the potential distribution of habitat using the produced binary maps of the studied species under different climate scenarios, the tendency for the geographical shift in the species distribution was analyzed. The main steps used in this study are shown in the provided flow chart (Fig. 2).

## Results

### Model performance and variables’ contribution under current condition

**Fig. 3:**
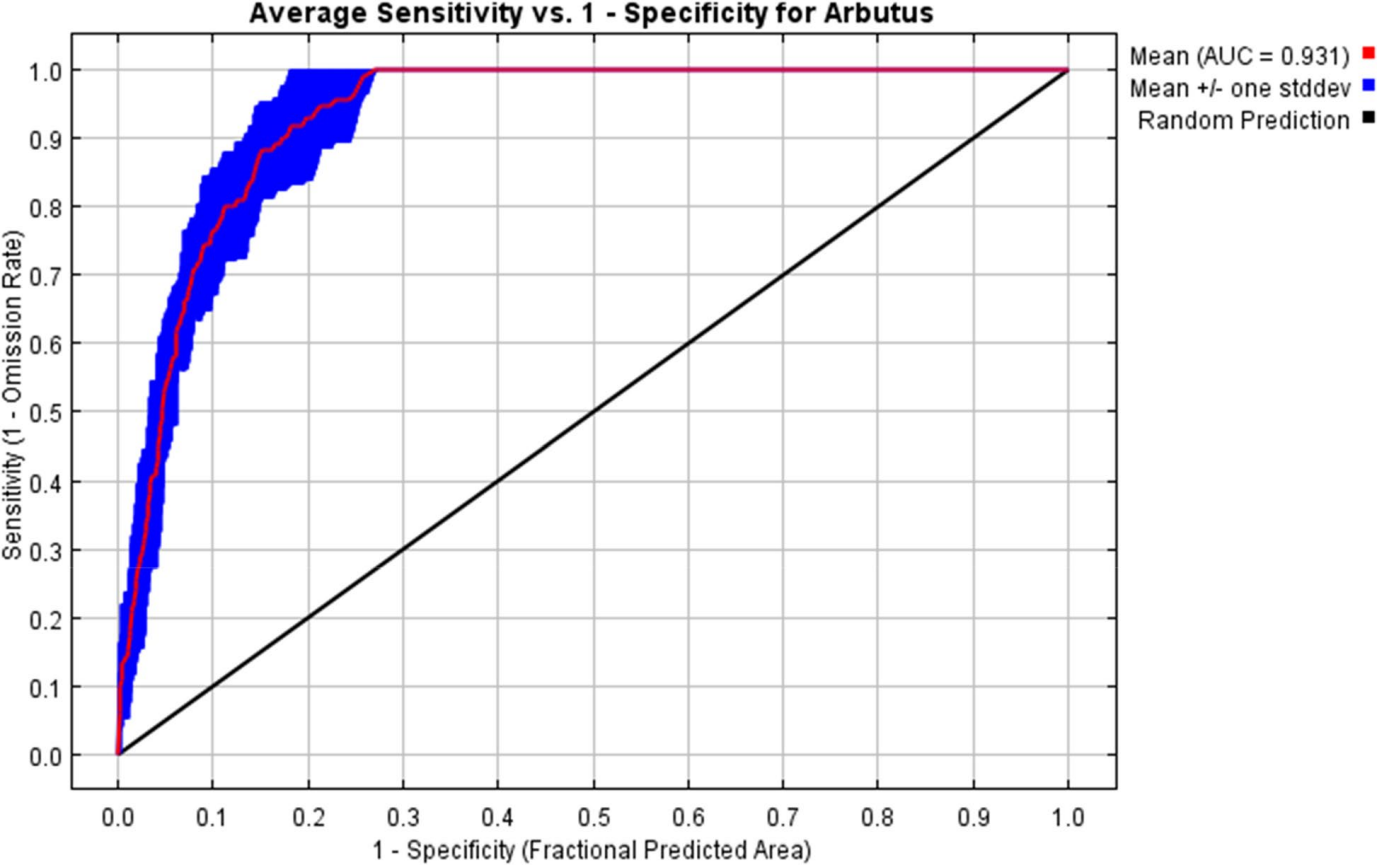
The receiver operating characteristics (ROC) curve for *Arbutus pavarii*. The red curve shows the mean response, and the blue margins are + / − one standard deviation calculated over 15 replicates

**Fig. 4:**
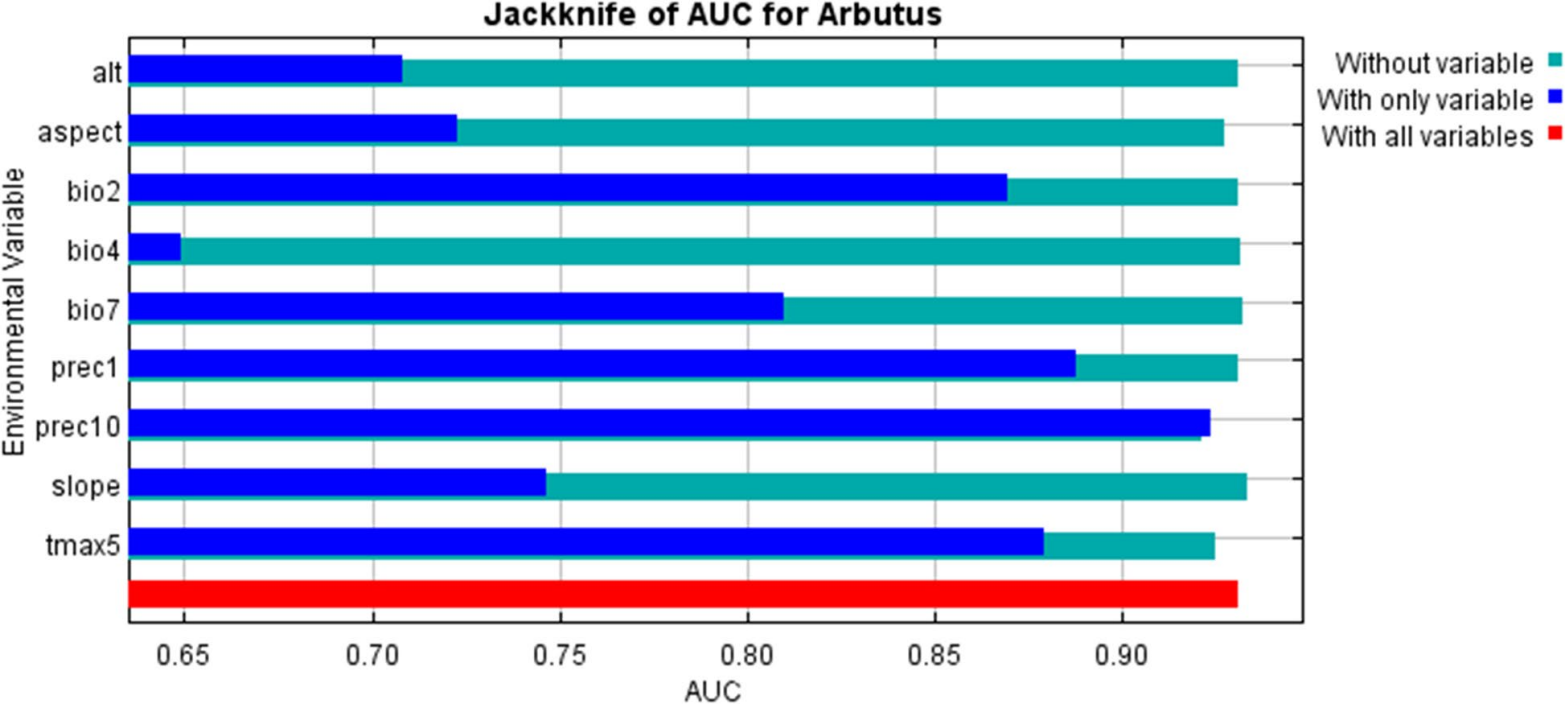
The results of the jackknife test of the relative importance of predictor variables

Our models showed high levels of predictive performances with values of AUC (training, 0.962 ± 0.001; test, 0.934 ± 0.009) (Fig. 3). The Jackknife test results of variables’ contribution to distribution modeling of *A. pavarii* are indicated in Fig. 4 and Table 1. Environmental predictors that exhibited the highest mean contributions were the precipitation in October (prec10), Mean Diurnal Range (Mean of monthly (max temp – min temp, bio2), and Annual Mean Temperature (bio1). Maximum temperature in May (tmax5), aspect, and temperature Annual Range (P5-P6) (bio7) provided high gains (> 2) to the model, indicating that these variables have the most useful information by themselves than the rest of the variables. Considering permutation importance, prec10 and tmax5 were the key environmental predictors that influenced the future distribution of *A. pavarii* (Table 1).

**Table 1:** Estimates of average contribution percent and permutation importance of the environmental variables used in MaxEnt modeling of *Arbutus pavarii*

Variables	Contribution percent	Permutation importance
prec10	67.64	71.52
bio2	9.48	3.6
prec1	7.82	3.82
tmax5	4.6	11.14
Aspect	4.48	2.4
bio7	4.12	3.56
Slope	1.1	0.96
bio4	0.54	2.3
Alt	0.16	0.74

**Fig. 5:**
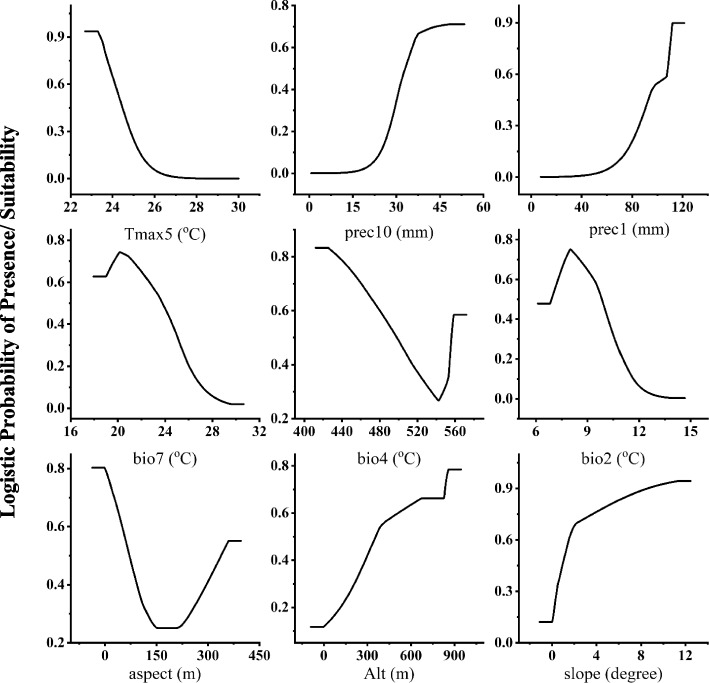
Response curves of nine environmental predictors used in the ecological niche model for *A. pavarii.* For abbreviations, See Table 2

The response curves of nine variables to *A. pavarii* habitat suitability are shown in Fig. 5. While considering the probability of temperature variables, bio2 of *A. pavarii* was 6.8–13.9 °C, whereas the Temperature Seasonality (bio4) ranged from 4.25 °C to 5.58 °C. In addition, bio7 varied from 19 to 29.6, whereas tmax5 varied from 23.29 to 27.22°C. Moreover, the precipitation in January (prec1) was 16.9 to 120 mm per year, while the habitat suitability also increased when the precipitation in October (prec10) was 11.7 to 49 mm per year. The suitable elevation, aspect, and slope ranges of *A. pavarii* were 5.90 to 560 m, 0.5 to 360 degrees, and 0.07 to 11.3%, respectively.

**Table 2:** The environmental variables for modeling the potential distribution of *A. pavarii* in the present study. Problems related to collinearity were avoided by removing variables with variance inflation factor (VIF) values > 5. The highlighted variables were selected through a multi-collinearity test and were used in modeling

Variables	Code/Unit	Source	VIF
Mean diurnal range (max. temp – min. temp)	bio2 (°C)	WorldClim	2.22
Temperature seasonality (SD × 100)	bio4 (°C)	WorldClim	2.17
Temperature annual range (Bio5-Bio6)	bio7 (°C)	WorldClim	4.09
Precipitation in January	prec1 (mm)	WorldClim	1.99
Precipitation in October	prec10 (mm)	WorldClim	2.39
Maximum Temperature in May	tmax5 (°C)	WorldClim	1.35
Elevation	Alt (m)	WorldClim	2.92
Aspect	aspect (degree)	Derived from elevation	4.30
Slope	Slope (%)	Derived from elevation	4.55

**Fig. 6:**
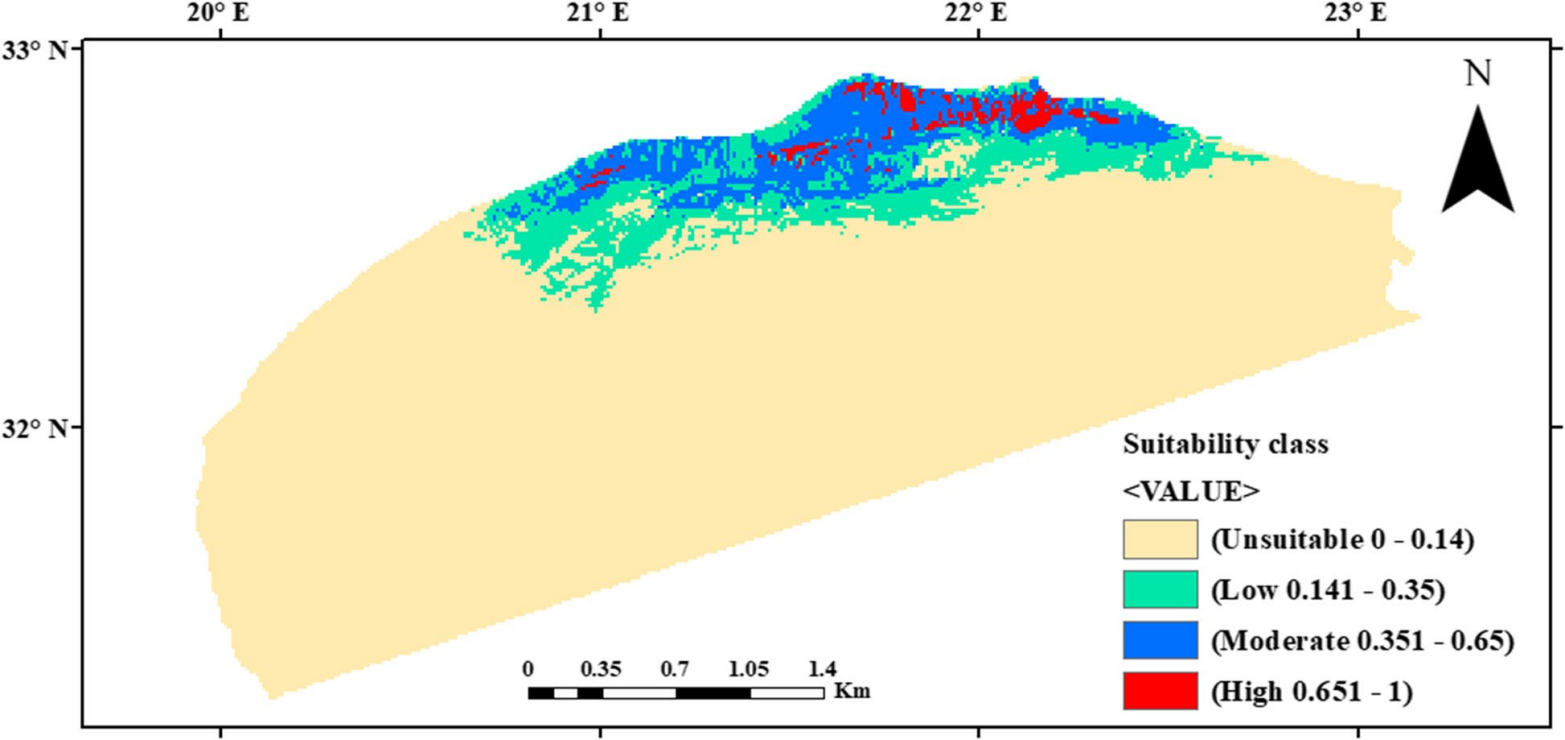
Map for potential current habitat suitability of *Arbutus pavarii* according to the occurrence records in Al-Jabal Al-Akhdar. The habitat suitability classes include unsuitable (0–14), low potential (0.141–0.35), moderate potential (0.351–0.65), and high potential (0.651–1.0)

The potential distribution map of *A. pavarii* in Al-Jabal Al-Akhdar is displayed in Fig. 6. Out of 42,118 km^2^ of the whole study area, 35,402 km^2^ (83.98% of the total area) was unsuitable for *A. pavarii*; the remaining 6716 km^2^ was divided into 3625 km^2^ (8.60% of the whole study area) with a low potential distribution, 2657 km^2^ (6.30% of the total area) with a moderate potential and only 434 km^2^ (1.03% of the total area) with the highest probability of suitable ecological conditions. Most probably suitable habitats (≥ 0.651) lie in the northeastern part of the AAR.

### Impacts of future climate change on *A. pavarii* distribution

**Fig. 7:**
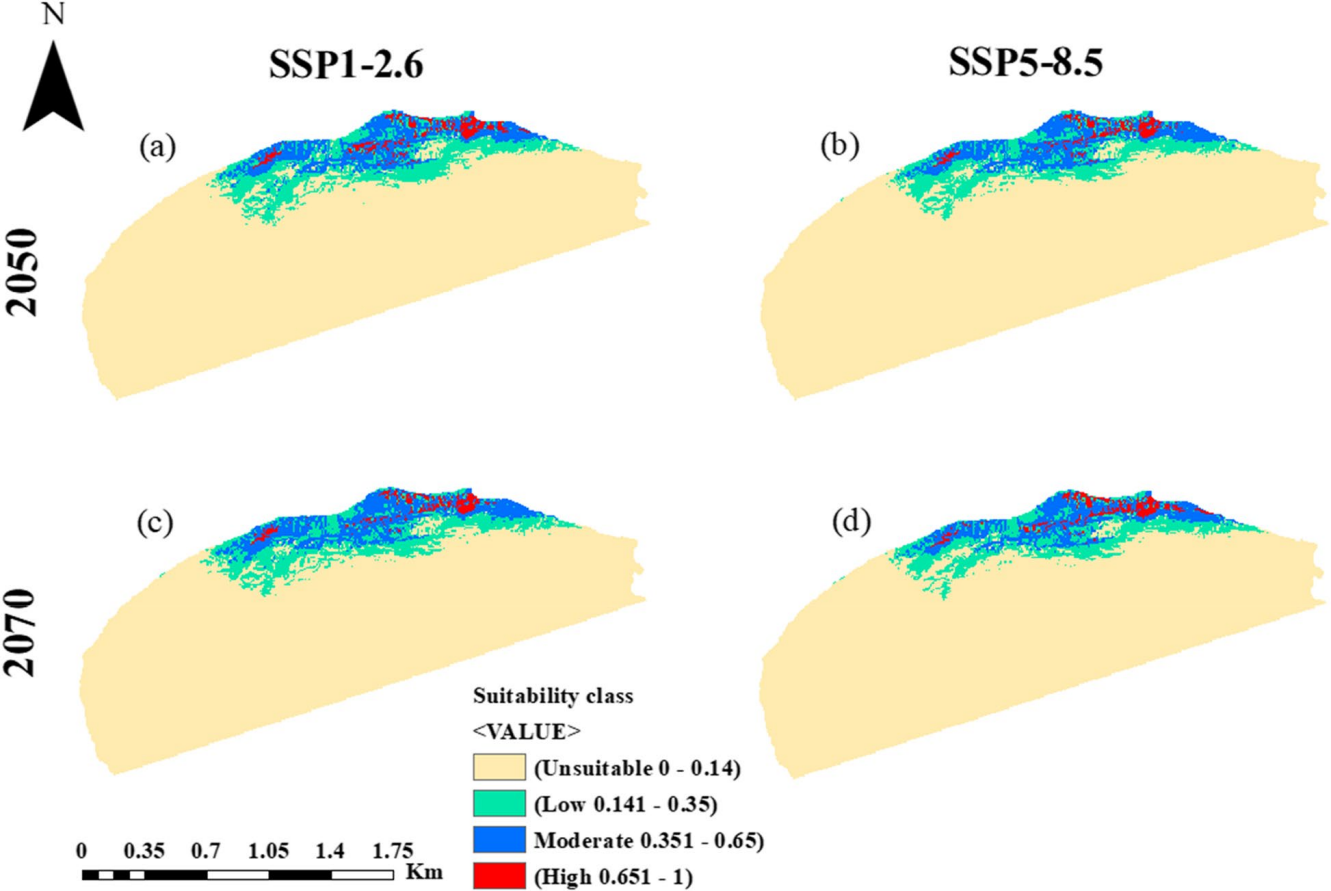
Ecological niche modeling of *Arbutus pavarii* based on the predicted climate change for 2050s (**a** and **b**) and 2070s (**c** and **d**) at two global warming scenarios: SSP1-2.6 (**a** and **c**) and SSP5-8.5 (**b** and **d**)

**Fig. 8:**
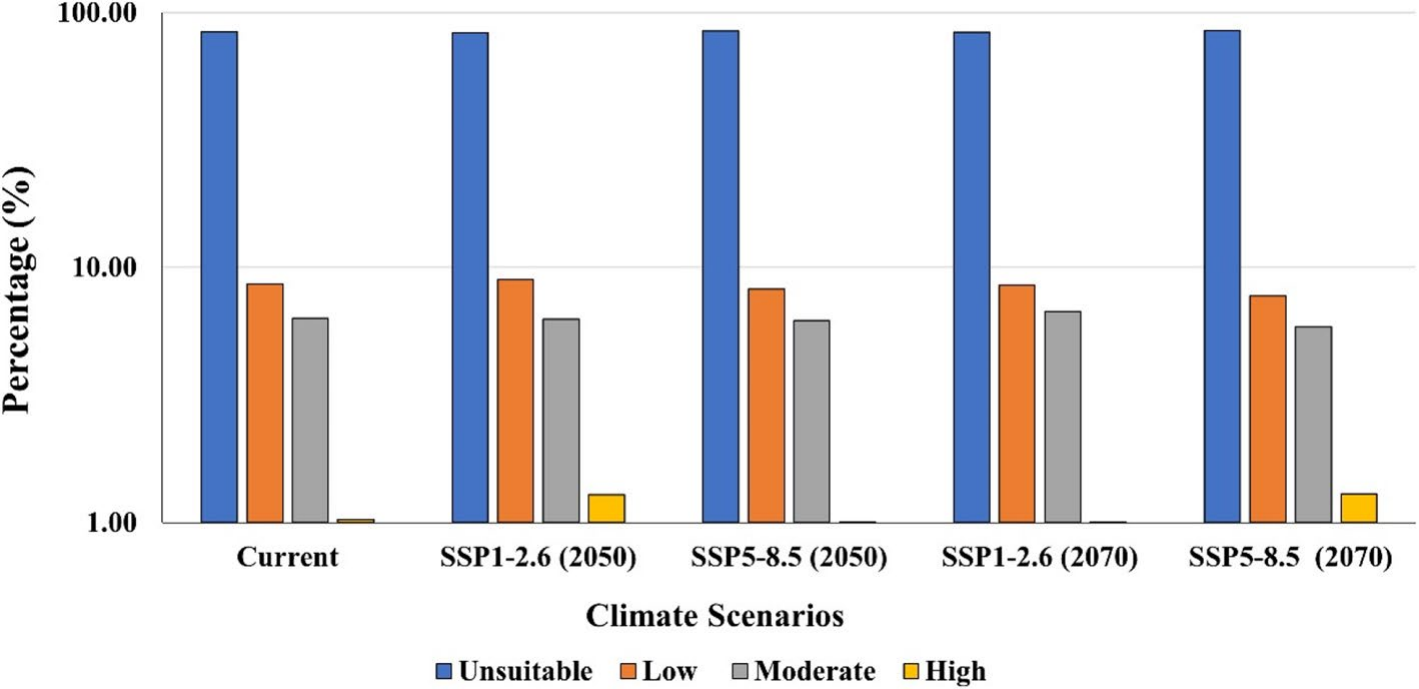
Predicted range changes (km^2^) for *Arbutus pavarii* distribution for 2050s and 2070s at two global warming scenarios SSP1-2.6 and SSP5-8.5 as compared with the potential current distribution

The potential impacts of future climate change on the distribution of *A. pavarii* under SSP1-2.6 and SSP5-8.5Shared Socio-economic Pathways are presented in Figs. 7 and 8 and Table 3. The model outcomes revealed that the suitability of habitats under the future climate scenarios is larger than the current projection under lower SSPs during the periods 2050s and 2070s. In contrast, the higher SSPs showed a loss in the suitability area during the two periods, 2050s and 2070s.

**Table 3:** Predicted range changes (km^2^) for *Arbutus pavarii* distribution for 2050s and 2070s at two global warming scenarios SSP1-2.6 and SSP5-8.5 as compared with the potential current distribution

**Suitability class**	Area & Percentage	**Current**	**HadGEM3-GC31-LL**			
			**2050s**		**2070s**	
			**SSP1-2.6**	**SSP5-8.5**	**SSP1-2.6**	**SSP5-8.5**
**Unsuitable**	km^2^	35,402	35,195	35,666	35,315	35,895
	(%)	83.98	83.49	84.60	83.77	85.15
**Low**	km^2^	3625	3773	3461	3590	3256
	(%)	8.60	8.95	8.21	8.52	7.72
**Moderate**	km^2^	2657	2645	2603	2829	2459
	(%)	6.30	6.27	6.17	6.71	5.83
**High**	km^2^	434	543	426	422	546
	(%)	1.03	1.29	1.01	1.00	1.30

By 2050s under SSP1-2.6, the expected suitable habitats for *A. pavarii* (≤ 0.14) within the AAR will increase by 8.95% and 1.29% for low and high suitability habitats as a result of climate change. However, all classes of habitat suitability of *A. pavarii* will decrease under SSP5-8.5. By the 2070s under SSP1.26, the low 8.52% and moderate 6.71% suitability classes will increase while the high suitability habitat will decrease. At SSP5-8.5 (2070), the low (7.72%) and moderate (5.83%) habitat suitability will decrease, while the high suitability habitat (1.30%) will increase.

**Fig. 9:**
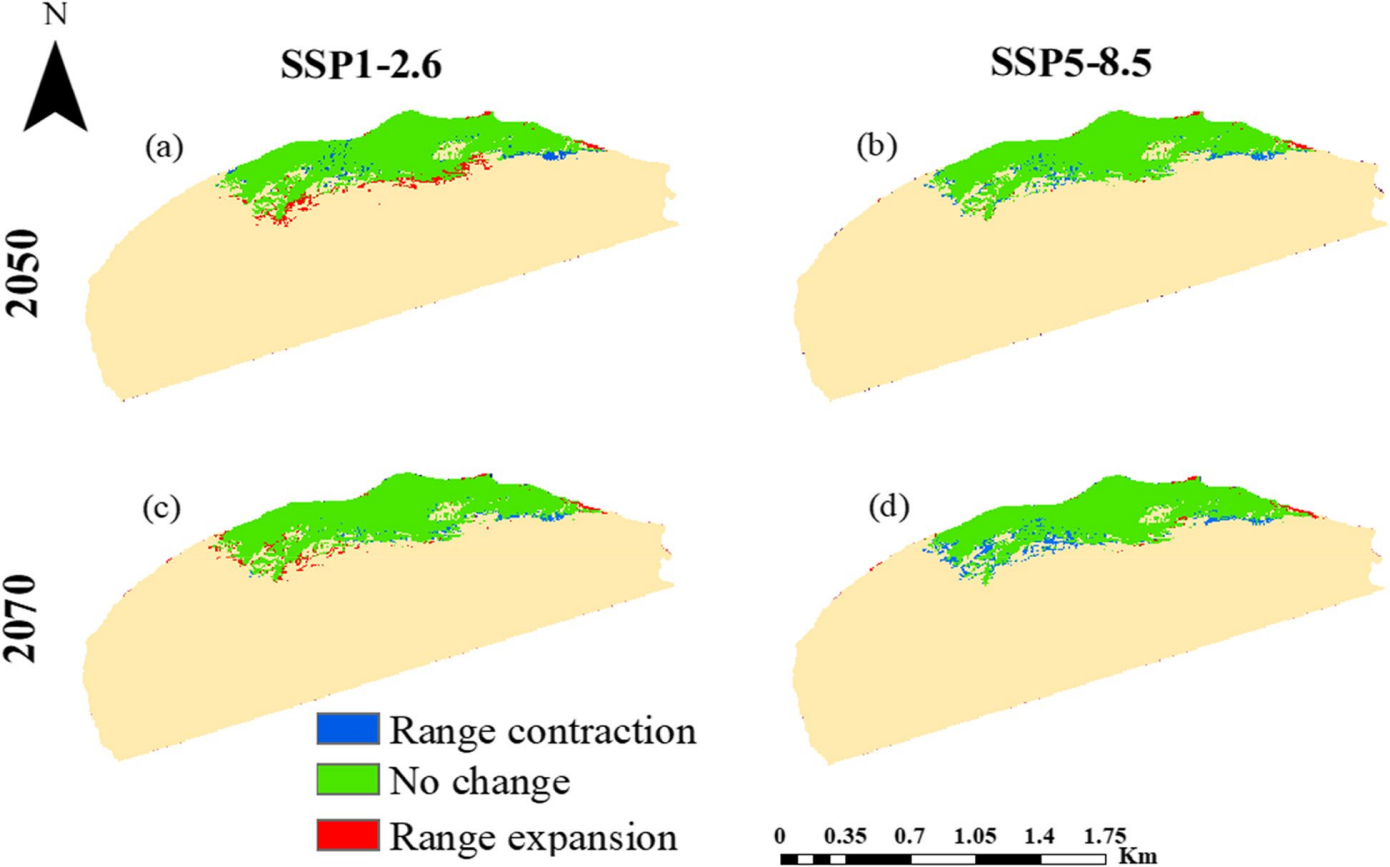
Spatial changes of *Arbutus pavarii* under climate change scenarios resulting from the MaxEnt model. The change in distribution between current and future climate conditions of the HadGEM3-GC31-LL general climate model under SSP1-2.6 (**a** & **c**) and SSP5-8.5 (**b** & **d**) scenarios by the period 2050s (**a** & **b**) and 2070s (**c** & **d**)

The predicted shift in the distribution pattern of the *A. pavarii* based on the HadGEM3-GC31-LL general circulation model under two Shared Socio-economic Pathways (SSP1-2.6 and SSP5-8.5) for the 2050s and 2070s is shown in Fig. 9 and Table 4. The habitats of the current distribution will undergo contraction by 0.59, 0.81, 0.42, and 1.39%and expansion by 1.12, 0.23, 0.67, and 0.26% under SSP1-2.6 (2050s), SSP5-8.5 (2050s), SSP1-2.6 (2070s) and SSP5-8.5 (2070s), respectively. At SSP1-2.6, the suitable habitat expansion of the species will be towards the southern borders of Al-Jabal Al-Akhdar in 2050 but along its southern western borders in 2070.

**Table 4:** The percentage of persistence, expansion, and contraction in the range of *Arbutus pavarii* predicted the MaxEnt model based on the HadGEM3-GC31-LL general climate model under the scenarios SSP1-2.6 and SSP5-8.5 during the period 2041–2060 and 2060–2080

Suitability class	Area & Percentage	HadGEM3-GC31-LL			
		**2050s**		**2070s**	
		**SSP1-2.6**	**SSP5-8.5**	**SSP1-2.6**	**SSP5-8.5**
**Range contraction**	km^2^	249	342	179	584
	(%)	0.59	0.81	0.42	1.39
**No change**	km^2^	6467	6374	6537	6132
	(%)	15.34	15.12	15.51	14.55
**Range expansion**	km^2^	473	95	283	108
	(%)	1.12	0.23	0.67	0.26

## Discussion

### Model performance and species response to environmental variables

In our study, we applied the MaxEnt model to forecast how future impacts of climate change will influence the narrow geographic occurrence of A. *pavarii* in the AAR based on different scenarios of climate change. With an AUC value > 0.93 for the current projection, a highly accurate model was developed using the selected environmental variables. In line with Elith et al. [18], the model performed well and accurately predicted the species' absence sites in the present study. The model evaluated the correlation between the species response and the environmental factors. It is obvious from the results that precipitation in October, aspect, and maximum temperature (mainly tmax5) are the factors that drive the occurrence of *A. pavarii* in its current location. Meanwhile, elevation has no considerable significant effect on the distribution of *A. pavarii*. Despite Kabiel et al. [39] reporting the great positive relationship between the growth of *A. pavarii* in the AAR and increasing in elevation, in this model elevation was not the most significant variable controlling the growth of its population. The resulting response curves and the potential current habitat suitability confirm this fact and the lands in the northeast direction of the study area where elevation is > 450 m a.s.l are the most suitable habitats for the species. Moreover, the high temperature associated with drought harmed the reproductive fitness of the plant. This is in agreement with the results of Larcher [40] who demonstrated the negative effect of high temperature on the growth of *Arbutus* spp. The heavy precipitation in the winter is one of the characteristic features of the study area [2]. The precipitation in October (the initiation of a new vegetative cycle) and January (winter) has a significant positive effect on the survival of the species. The key role of precipitation, the temperature of the air, and elevation in the determination of the distribution of plants were reported for the endemic species, *Rosa arabica* that grown in the harsh environmental conditions in the St. Catherine protectorate, Egypt [3, 47].

According to Al-Zeni and Bayoumi [7], *A. pavarii* grows in many habitats, particularly along the ground and mountainous slopes along the northern and southern directions, which confirms our findings. The distribution of the species under current conditions shows that most of the lands in the AAR are not suitable for the occurrence of species. Furthermore, the current absence of *A. pavarii* from many sites in the AAR may be attributed to its low fecundity as reported by Mosallam et al. [43].

The potential reasons behind the species’ current presence in the modeled suitable areas are the assemblage of favorable conditions at an elevation > 450 m with enough precipitation and low tmax that reduce the drought stress on the species. Moreover, the current modeled suitable areas are characterized by low human interferences and presence of the species at many slopes and difficult accessible lands.

### Impacts of climate change on habitat suitability of *A. pavarii*

According to MaxEnt modeling results for the future scenarios (the 2050s and 2070s), the species' suitability of habitat will face fluctuation in its distribution in near and far future terms, with mostly more decrease by raising the air temperature. The expansion in the distribution range of *A. pavarii* will be high under SSP1-2.6 scenarios in the 2050s and 2070s compared to SSP5-8.5. In contrast, under the higher emissions scenario SSP5-8.5, the range contraction of the species will be high. This may emphasize the negative impact of high temperatures on the presence of the species as a driving factor for the growth of *A. pavarii* besides the other contributing factors e.g. precipitation. The elevation and precipitation range of the future suitable distribution habitats (expansion areas) will be 257–490 m a.s.l. and 300 to 400 mm year ^−1^, respectively. The suitable aspect value for the distribution of the species under the future scenarios’ projections should be considered. In contrast, the areas where the distribution of *A. pavarii* will be contracted are characterized by low elevation (217–279 m a.s.l.) and precipitation range (200 to 250 mm year ^−1^). Nijland et al. [46] found a positive association between *Arbutus unedo* and precipitation and a negative relationship with summer heat. It seems that under the low emission scenario (SSP1-2.6), the expansion of *A. pavarii* will be toward the western south of the studies area and some peripheral spots at the eastern section of the AAR. In contrast, the contraction of its distribution will be in some of the current suitable areas. Under the high emissions scenario (SSP5-8.5), most of the retraction will be on the southern eastern and western parts of the study area.

The influence of altitude and other environmental variables on endangered and endemic plant species has been reported by many authors. In some cases, the highlands in semi-arid environments with low precipitation led to a decline in the plant growth and distribution of plant species such as *Juniperus phoenicea* [13, 22] and *Moringa peregrina* [23]. In contrast, Arvin et al. [8] found that cool foothills with high elevation (1670–2000 m a.s.l.) associated with annual precipitation > 250 mm, and annual mean temperature < 15 °C are suitable areas for *Thymus daenensis*. Hosseini et al. [36] studied the effect of future climate change on two *Thymus* species in Iran. They found that the distribution of *Thymus daenensis* was most significantly influenced by the mean temperature of the warmest quarter (bio10). For *Thymus kotschyanus*, the main determining factor was slope %. The same was reported by Erfanian et al. [21] for three plant species that thrive at narrow elevations in the Khorassan-Kopet Dagh floristic province, Iran. In Northwest China, Zhang and Zhao [57] found that the primary factors affecting the hot regions of rare and endangered plants are temperature, precipitation, and altitude. This may indicate the high sensitivity of the rare and endemic species with narrow ecological distribution to environmental factors.

Conservation of rare and endangered plant species is critical for biodiversity [41]. The role of *A. pavarii* in the ecosystem is valuable and provides many ecosystem services and goods as mentioned in the introduction section. This entails the conservation of this species and preserving its current habitats. Any extinction of this species means losing a unique genetic resource and plant species only grow in Libya.

## Conclusions

According to the results of the present study, precipitation in October, aspect, and maximum temperature (mainly tmax5) are the main controlling factors for the occurrence of *A. pavarii* in its current location. The highlands at 257–490 m a.s.l with 300 to 400 mm year ^−1^ will be the most suitable habitats for the species in the future. The suitable habitats for *A. pavarii* will experience both expansions and contractions. Generally, the current highly suitable habitat of *A. pavarii* will contract in the future compared to the low and moderate suitable areas. With the expected increase in air temperature coupled with low reproductive fitness, *A. pavarii* is likely to have more difficulty surviving and spreading naturally. Additional stressors including increased grazing, seeds, and timber harvesting make it difficult for *A. pavarii* to survive in its existing suitable habitats. Further studies on the ecophysiology of the adult tree of *A. pavarii* under drought stress and the long-term growth-climate relationship of the species are crucial to understanding whether or not *A. pavarii* is flexible enough to adapt to expected global warming. We recommend the in-situ conservation of *A. pavarii* and its cultivation in the projected high and moderate habitats. Local community engagement may be beneficial in any conservation program for this species.

## Supplementary Information

Supplementary Material 1.

## Data Availability

Data is provided within the supplementary information files.
