# Supplementary material for: The sensitivity and response of the threatened endemic shrub Arbutus pavarii to current and future climate change
Source: BMC Ecol Evol. 2025 Apr 24;25:36. doi: 10.1186/s12862-025-02370-2 (PMC12020073; doi:10.1186/s12862-025-02370-2)
Supplement: Supplementary file 1 — Supplementary Material 1. [file 12862_2025_2370_MOESM1_ESM.docx]

**Supplementary Materials**

Table S1. Environmental variables used in the study (sources: <http://worldclim.org/version2>.1 and <https://www.usgs.gov>)

| Abbreviation | Variables | Units |
| --- | --- | --- |
| Climatic/Bioclimatic variables | | |
| bio1 | Annual mean temperature | °C |
| bio2 | Mean diurnal range (mean of monthly (max temp – min temp)) | °C |
| bio3 | Isothermality (P2/P7) ×100 |  |
| bio4 | Temperature seasonality (standard deviation×100) |  |
| bio5 | Max temperature of warmest Month | °C |
| bio6 | Min temperature of coldest Month | °C |
| bio7 | Temperature annual range (P5-P6) | °C |
| bio8 | Mean temperature of wettest quarter | °C |
| bio9 | Mean temperature of driest quarter | °C |
| bio10 | Mean temperature of warmest quarter | °C |
| bio11 | Mean temperature of coldest quarter | °C |
| bio12 | Annual precipitation | mm |
| bio13 | Precipitation of wettest Month | mm |
| bio14 | Precipitation of driest Month | mm |
| bio15 | Precipitation of seasonality (Coefficient of Variation) |  |
| bio16 | Precipitation of wettest quarter | mm |
| bio17 | Precipitation of driest quarter | mm |
| bio18 | Precipitation of warmest quarter | mm |
| bio19 | Precipitation of coldest quarter | mm |
| Prec | Precipitation | mm |
| prec1 | Precipitation in January | mm |
| prec2 | Precipitation in February | mm |
| prec3 | Precipitation in March | mm |
| prec4 | Precipitation in April | mm |
| prec5 | Precipitation in May | mm |
| prec6 | Precipitation in June | mm |
| prec7 | Precipitation in July | mm |
| prec8 | Precipitation in August | mm |
| prec9 | Precipitation in September | mm |
| prec10 | Precipitation in October | mm |
| prec11 | Precipitation in November | mm |
| prec12 | Precipitation in December | mm |
| tmax | Maximum temperature | °C |
| tmax1 | Maximum temperature in January | °C |
| tmax2 | Maximum temperature in February | °C |
| tmax3 | Maximum temperature in March | °C |
| tmax4 | Maximum temperature in April | °C |
| tmax5 | Maximum temperature in May | °C |
| tmax6 | Maximum temperature in June | °C |
| tmax7 | Maximum temperature in July | °C |
| tmax8 | Maximum temperature in August | °C |
|  |  |  |

**Table S1.** **(Continued)**

| Abbreviation | Variables | Units |
| --- | --- | --- |
| Climatic/Bioclimatic variables | | |
| tmax9 | Maximum temperature in September | °C |
| tmax10 | Maximum temperature in October | °C |
| tmax11 | Maximum temperature in November | °C |
| tmax12 | Maximum temperature in December | °C |
| tmin | Minimum temperature | °C |
| tmin1 | Minimum temperature in January | °C |
| tmin2 | Minimum temperature in February | °C |
| tmin3 | Minimum temperature in March | °C |
| tmin4 | Minimum temperature in April | °C |
| tmin5 | Minimum temperature in May | °C |
| tmin6 | Minimum temperature in June | °C |
| tmin7 | Minimum temperature in July | °C |
| tmin8 | Minimum temperature in August | °C |
| tmin9 | Minimum temperature in September | °C |
| tmin10 | Minimum temperature in October | °C |
| tmin11 | Minimum temperature in November | °C |
| tmin12 | Minimum temperature in December | °C |
| tavg | Average temperature | °C |
| tavg1 | Average temperature in January | °C |
| tavg2 | Average temperature in February | °C |
| tavg3 | Average temperature in March | °C |
| tavg4 | Average temperature in April | °C |
| tavg5 | Average temperature in May | °C |
| tavg6 | Average temperature in June | °C |
| tavg7 | Average temperature in July | °C |
| tavg8 | Average temperature in August | °C |
| tavg9 | Average temperature in September | °C |
| tavg10 | Average temperature in October | °C |
| tavg11 | Average temperature in November | °C |
| tavg12 | Average temperature in December | °C |
| Topographic variables | | |
| Alt | Elevation | m |
| Slope | Slope | % |
| Aspect | Aspect | Degree |
|  |  |  |
